# Uptake Dynamics of Ionic and Elemental Selenium Forms and Their Metabolism in Multiple-Harvested Alfalfa (*Medicago sativa* L.)

**DOI:** 10.3390/plants10071277

**Published:** 2021-06-23

**Authors:** Zoltán Kovács, Áron Soós, Béla Kovács, László Kaszás, Nevien Elhawat, Nóra Bákonyi, Mutasem Razem, Miklós G. Fári, József Prokisch, Éva Domokos-Szabolcsy, Tarek Alshaal

**Affiliations:** 1 Agricultural Botany, Plant Physiology and Biotechnology Department, University of Debrecen, Böszörményi Str. 138, 4032 Debrecen, Hungary; kovacs.zoltan@agr.unideb.hu (Z.K.); kaszas.laszlo@agr.unideb.hu (L.K.); nevienadelismailelhawat@azhar.edu.eg (N.E.); nbakonyi@agr.unideb.hu (N.B.); fari@agr.unideb.hu (M.G.F.); szabolcsy@agr.unideb.hu (É.D.-S.); 2 Institute of Food Science, University of Debrecen, Böszörményi Str. 138, 4032 Debrecen, Hungary; soosaron@gmail.com (Á.S.); kovacsb@agr.unideb.hu (B.K.); mutasemrazem@gmail.com (M.R.); 3 Department of Biological and Environmental Sciences, Faculty of Home Economic, Al-Azhar University, Tanta 31732, Egypt; 4 Institute of Animal Science, Biotechnology and Nature Conservation, University of Debrecen, Böszörményi Str. 138, 4032 Debrecen, Hungary; jprokisch@agr.unideb.hu; 5 Soil and Water Science Department, Faculty of Agriculture, Kafrelsheikh University, Kafr El-Sheikh 33516, Egypt

**Keywords:** agronomic biofortification, alfalfa, red elemental Se, selenomethionine, uptake dynamics

## Abstract

A pot experiment, under greenhouse conditions, was carried out aiming at investigating the agronomic biofortification of alfalfa (*Medicago sativa* L.) with Se and monitoring the Se uptake and accumulation dynamics within four consecutive harvests within the same growing season. Two ionic Se forms, i.e., sodium selenate (Se (VI)) and sodium selenite (Se (IV)), were applied once at a rate of 1, 10, and 50 mg kg^−1^ (added on Se basis), while 10 and 50 mg L^−1^ of a red elemental Se (red Se^0^) were used; all Se treatments were added as soil application. Application of Se (VI) at the rate of 50 mg kg^−1^ was toxic to alfalfa plants. The effect of Se forms on Se accumulation in alfalfa tissues, regardless of the applied Se concentration, follows: Se (VI) > Se (IV) > red Se^0^. The leaf, in general, possessed higher total Se content than the stem in all the treatments. The accumulation of Se in stem and leaf tissues showed a gradual decline between the harvests, especially for plants treated with either Se (VI) or Se (IV); however, the chemically synthesized red Se^0^ showed different results. The treatment of 10 mg kg^−1^ Se (VI) resulted in the highest total Se content in stem (202.5 and 98.0 µg g^−1^) and leaf (643.4 and 284.5 µg g^−1^) in the 1st and 2nd harvests, respectively. Similar tendency is reported for the Se (IV)-treated plants. Otherwise, the application of red Se^0^ resulted in a lower Se uptake; however, less fluctuation in total Se content between the four harvests was noticed compared to the ionic Se forms. The Se forms in stem and leaf of alfalfa extracted by water and subsequently by protease XIV enzyme were measured by strong anion exchange (SAX) HPLC-ICP-MS. The major Se forms in our samples were selenomethionine (SeMet) and Se (VI), while neither selenocysteine (SeCys) nor Se (IV) was detected. In water extract, however, Se (VI) was the major Se form, while SeMet was the predominant form in the enzyme extract. Yet, Se (VI) and SeMet contents declined within the harvests, except in stem of plants treated with 50 mg L^−1^ red Se^0^. The highest stem or leaf SeMet yield %, in all harvests, corresponded to the treatment of 50 mg L^−1^ red Se^0^. For instance, 63.6% (in stem) and 38.0% (in leaf) were calculated for SeMet yield % in the 4th harvest of plants treated with 50 mg L^−1^ red Se^0^. Our results provide information about uptake and accumulation dynamics of different ionic Se forms in case of multiple-harvested alfalfa, which, besides being a good model plant, is an important target plant species in green biorefining.

## 1. Introduction

Forage plants contribute to mitigating the negative environmental effects of intensified agriculture resulting in enhanced soil health and fertility, increased carbon sequestration, root disease management in cropping systems, increased biodiversity. In addition, forages play pivotal role in crop-livestock farming systems [1]. Around 60 different plants of *Leguminosae* family are known and cultivated as sources of forage for animals. Among them alfalfa (*Medicago sativa* L.) is the most frequent [2]. The global yield of alfalfa is more than 400 million metric tons per year [3]. Alfalfa originated from the Middle East; however, alfalfa cultivation nowadays is widespread around the world. Alfalfa possesses a deep root system capable to reach deep water supplies and tolerating drought [4]. Moreover, its dense root system enriches the soil organic matter content, soil biological activity, provides physical protection against wind and water erosion also reduces soil erosion [5]. Alfalfa, a herbaceous perennial plant, can regrow from the buds located in the crown after dormant state caused by unfavorable growth conditions [6]. Green biomass of alfalfa can be harvested 3–7 times per vegetation season depending on the variety, temperature, and irrigation. The multiple-harvested green biomass can provide 2–3 metric ton ha^−1^ crude protein; in addition to being a good source of chlorophylls, carotenoids, vitamins such as vitamin C, E, and different forms of vitamin Bs. Among secondary metabolites several flavonoids, phenolic compounds, phytoestrogens and saponins could be identified in alfalfa shoot. Moreover, it contains valuable minerals such as Ca, Cu, Fe, Mg, Mn, P, Zn, Si [7]. 

One of the future challenges of agriculture is the depletion of several trace elements such as iron (Fe), zinc (Zn), iodine (I_2_), and selenium (Se) [8]. In addition to soil depletion, these minerals will also be valued in plant-based diets as they are typically found in less accessible amounts in plant-based foods than in animals, due to inhibitory substances. Selenium (Se) is one of the intensely studied microelements. Although Se is essential only for algae in the Plant Kingdom, several benefits to plant growth, particularly for those grown under biotic and/or abiotic stress, have been documented within many studies [5,9,10,11]. However, nowadays, the presence of an adequate concentration of Se in food and feed has become a necessity after stating its indispensability to humans and animals. For instance, for humans, the advised daily dose of Se is 55 µg day^−1^ with a tolerance limit of up to 400 µg day^−1^ as stated by the WHO and FAO [12], whereas higher doses of Se are required by animals. Beef cattle, for example, require 100 µg kg^−1^ (on dry matter basis), while dairy cows demand 300 µg kg^−1^ (on dry matter basis) [13]. In animals, more than 30 selenoproteins, including the well-known antioxidant enzyme glutathione peroxidase (GSH), have been identified for their crucial roles in production, disease protection, and fertility [14]. The concentration in the soil, plant uptake, and accumulation of Se are decisive in the Se level in food and feed. Because of its close connection Se in plant-based food and feed varies widely by geographical location [15]. Plants accumulated Se below 100 µg g^−1^ DW are known as non-accumulators, while hyperaccumulators plants are classified into two groups as follows: (1) secondary accumulators (100–1000 µg Se g^−1^ DW) and (2) primary accumulators (1000–15,000 µg Se g^−1^ DW) [16,17]. Mikkelsen et al. [18] reported that alfalfa plants including their root system accumulated 948 mg Se kg^−1^ upon exposure to 1 mg L^−1^ Se (VI). However, lower Se contents were reported in alfalfa grown on 900 μM Se, added as sodium selenate in the nutrient solution, for 60 days recording 4.37 mg kg^−1^ (in leaves), 3.75 mg kg^−1^ (in stem), and 6.30 mg kg^−1^ (in root) [19]. 

In Se-poor areas, the exogenous application of Se is necessary. Phytofortification/biofortification can offer an opportunity to improve the bioavailable concentrations of microelement in edible portions of crop plants and through them into the food chain. Fortification can be achieved by agronomic intervention or using traditional breeding practices and/or genetic engineering [9]. Regarding agronomic fortification, it is worth noting the nanotechnology, which is a rapidly developing technique for targeted and precise micronutrient fertilizing in agriculture [10,20]. Biofortification is a term that can be approached from two perspectives, depending on whose interests are in focus. On the one hand, crops and forages can be fortified with Se to avoid the suboptimal micronutrient level in consumers including livestock and/or humans. Hence, insufficient intake of Se in humans is correlated with low protection against the oxidizing agents and increased risk of different types of cancer and cardiovascular diseases [21]. Moreover, decreased productive and reproductive performance is observed in livestock due to suboptimal levels of dietary Se [22,23]. Seboussi et al. [24] showed that Se-enriched forages (timothy and alfalfa silage) had more bioavailable Se form in the diet of cows than inorganic Se form and it was more effective in increasing milk and blood Se concentrations. Se-fortified alfalfa hay improved vaccination responses and subsequent growth and survival of beef calves in the feedlot; furthermore, it promoted the accumulation of Se and antibodies in the colostrum of calves [25]. 

On the other hand, from plant aspects, the importance of Se biofortification is a widely researched area. Selenium, like several stressors, including certain toxins, biostimulants, or non-essential elements elicits a biphasic dose-response. In other words selenium induces dose-dependent stimulation/inhibition in plants. This phenomenon, called also hormesis, facilitates the acclimatization of plants in new or changing environments, and it can be a key factor in evolutionary processes [26]. For instance, Se in low concentration range enhances the plant growth and mitigates the harmful effects of abiotic environmental factors depending on the plant species [27]. Most studies related to Se fortification focus on the plant biological effects of the canopy and/or fruit that are harvested once during the experiment [28]. From this approach, alfalfa is a special non-selenium accumulator forage crop. As a multi-harvested plant, it can be a good model to compare the Se uptake and translocation dynamics of different chemical forms in newly regrown leafy shoots. At the same time, alfalfa has a great importance in animal feeding. Hence, to investigate the Se uptake, translocation, accumulation, and conversion dynamics in re-growing green biomass provide useful information in developing a balanced forage-fortification method.

Our objectives are to compare the uptake of three different inorganic chemical forms of Se (i.e., Se (VI), Se (IV), and red Se^0^) by alfalfa. Further aim is to examine the translocation, accumulation dynamics of Se forms in green biomass during four consecutive harvests in the same growing season and the effects of these forms on the plant metabolism. Therefore, total Se content, Se speciation, stress response to Se application, and plant biometrics were measured.

## 2. Results

### 2.1. Selenium Accumulation in Alfalfa

#### 2.1.1. Total Se Content

**Figure 1:**
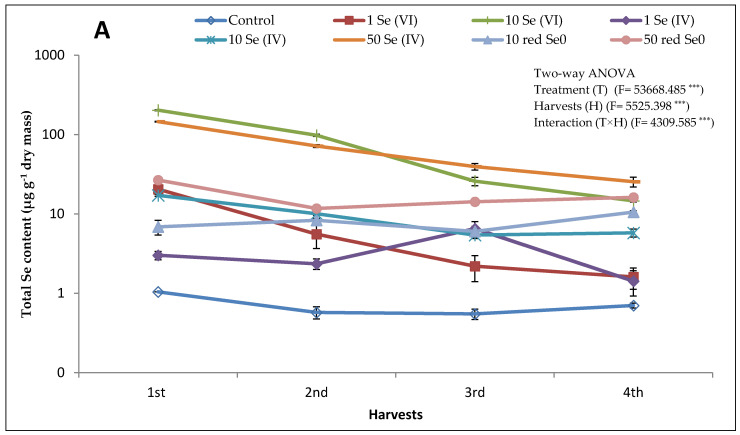

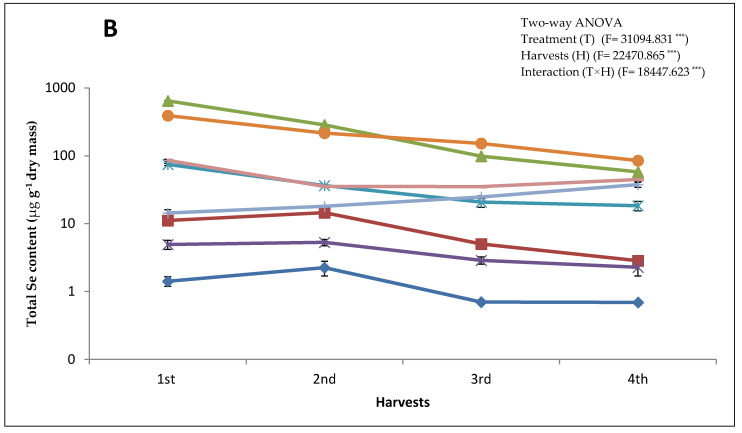
Total selenium (Se) content in alfalfa aboveground biomass (**A**: stem; **B**: leaves) fertilized with different Se forms (i.e., Se (VI), Se (IV) and red Se^0^) at different concentrations (1, 10, and 50 mg kg^−1^ for ionic forms and 10 and 50 mg L^−1^ for red Se^0^) within four consecutive harvests with 30-day intervals. Alfalfa plants that received 50 mg kg^−1^ Se (VI) died two weeks after seed germination. Data are means ± SD (*n* = 6). *** significant according to Tukey’s test (*p* ≤ 0.001).

The uptake and accumulation of total Se in alfalfa stem show a dose-response relationship regardless of the applied chemical form of Se and harvest time (Figure 1A). For instance, as much as 202.5 µg Se g^−1^ at the rate of 10 mg kg^−1^ Se (VI) added as soil application to as little as 20.5 µg g^−1^ at the rate of 1 mg kg^−1^ Se (VI) added as soil application in the first harvest. Moreover, in the 1st and 2nd harvests, the highest total Se content in stem (202.5 and 98.0 µg g^−1^, respectively) corresponded to the application of Se (VI) at the rate of 10 mg kg^−1^; while in the 3rd and 4th harvests, the application of 50 mg kg^−1^ Se (IV) resulted in the highest total Se content in stem (39.5 and 25.4 µg g^−1^, respectively). Moreover, total Se content in stem shows a linear reduction within the harvests where the 4th harvest displayed the lowest total Se content in most of the treatments. The accumulation of Se in the stem was significantly affected by the applied chemical form of Se; the Se (VI), regardless of its concentration, possessed the highest total Se content compared to the same concentration of Se (IV) and red Se^0^ (Figure 1A). A regular decrease in total Se content with the harvest time was noticed under the treatments of 1 and 10 mg kg^−1^ Se (VI) and 10 and 50 mg kg^−1^ Se (IV). For instance, total Se content in the 1st, 2nd, 3rd, and 4th harvest was 202.5, 98.0, 25.8, and 14.6 µg g^−1^ when alfalfa plants were grown on 10 mg kg^−1^ Se (VI). The total Se content in stem of plants treated with either 10 or 50 mg L^−1^ red Se^0^ was significantly lower compared to 10 mg kg^−1^ Se (VI) treatment in all the harvests, except in the 4th harvest where almost same contents were measured. The application of red Se^0^ showed a lower Se uptake compared to the Se (VI) and Se (IV); however, a slight fluctuation in the total Se content between the four harvests was noticed (Figure 1A,B). In the presence of 10 mg L^−1^ red Se^0^, the total Se content in the 1st harvest was 6.9 µg g^−1^, while in the 4th harvest was 10.5 µg g^−1^. Upon the application of 50 mg L^−1^ red Se^0^, a 26.8, 11.7, 14.2, and 16.2 µg g^−1^ of total Se was measured in the 1st, 2nd, 3rd, and 4th harvests, respectively (Figure 1A). 

Overall, evolution of the total Se content in alfalfa leaves differed slightly from stem (Figure 1B). For instance, the application of Se (VI) at the rate of 10 mg kg^−1^ resulted in the highest total Se content in leaves in the 1st and 2nd harvests, while the highest total Se content in the 3rd and 4th harvests corresponded to the 50 mg Se kg^−1^ Se (IV). In the treatment of 10 mg kg^−1^ Se (VI) and 50 mg kg^−1^ Se (IV) a linear reduction in total Se content was detected within the four harvests. However, the other treatments hesitantly affected the total Se content in leaves. Interestingly, upon the application of 10 mg L^−1^ red Se^0^, the total Se content in leaves increased gradually from the 1st harvest (14.3 µg g^−1^) to the 4th harvest (37.5 µg g^−1^). Despite the application of 50 mg L^−1^ red Se^0^ it did not show the same trend as the 10 mg L^−1^ red Se^0^, it exhibited a reduction in the total Se content from the 1st to the 3rd harvest then increased again in the 4th harvest. However, the reduction in the total Se content was 47.8% for the treatment of 50 mg L^−1^ red Se^0^ while for the 10 mg kg^−1^ Se (VI) and 50 mg kg^−1^ Se (IV) treatments was 91.0 and 78.3%, respectively.

#### 2.1.2. Selenium Forms 

**Figure 2:**
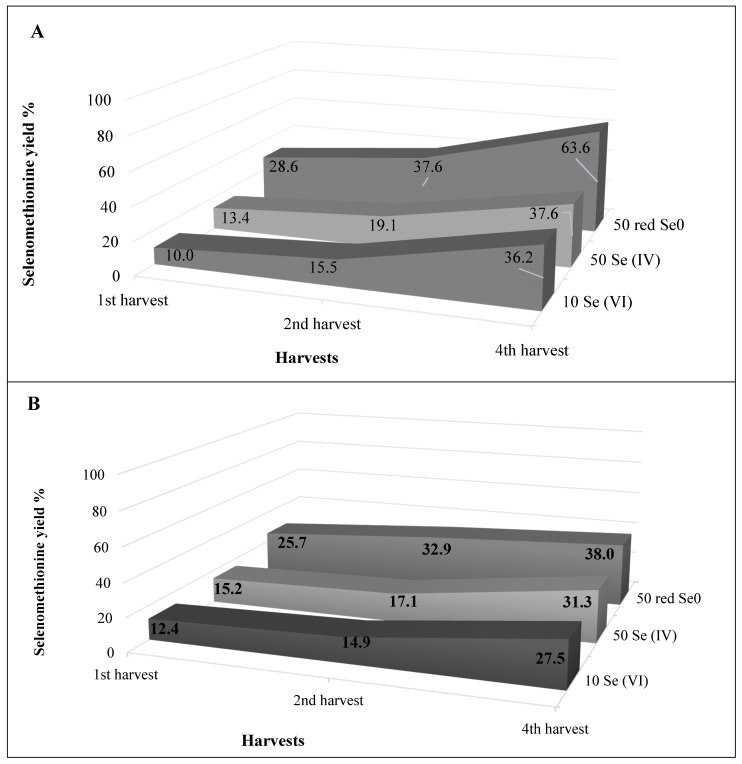
Selenomethionine (SeMet) yield % in alfalfa aboveground biomass (**A**: stem; **B**: leaves) fertilized with different Se forms (i.e., Se (VI), Se (IV), and red Se^0^) at different concentrations (10 and 50 mg kg^−1^ for ionic form and 50 mg L^−1^ for red Se^0^) within three harvests.

Selenium forms in alfalfa aboveground part were identified and quantified with the external calibration method from the 1st, 2nd, and 4th harvests. Regarding quality assurance, Se-enriched yeast (SELM-1) as a certified reference material was analyzed. The recovery % of Se from SELM-1 was 80.4% for SeMet. The Se speciation was carried out for the treatments with the highest Se concentration, i.e., 10 mg kg^−1^ Se (VI), 50 mg kg^−1^ Se (IV); and 50 mg L^−1^ red Se^0^, in the 1st, 2nd, and 4th harvests. Based on SAX HPLC-ICP-MS results, inorganic Se (VI) was the dominant peak in water-soluble stem and leaf fractions regardless of the applied Se treatment (Table 1 and Figure S1). However, Se (VI) content drastically declined from the 1st harvest to the 4th one in the treatments of 10 mg kg^−1^ Se (VI) and 50 mg kg^−1^ Se (IV). For instance, the stem Se (VI) concentration in the treatment of 50 mg kg^−1^ Se (IV) decreased from 89.0 µg g^−1^ (in the 1st harvest) to 5.16 µg g^−1^ (in the 4th harvest). Likewise, in leaves, Se (VI) content reduced from 336.0 (in the 1st harvest) to 20.5 µg g^−1^ (in the 4th harvest) in the treatment of 50 mg kg^−1^ Se (IV) (Table 1). The accumulation of Se in the form of Se (VI) in either stem or leaf of alfalfa was lower upon the application of 50 mg kg^−1^ Se (IV) or 50 mg L^−1^ red Se^0^ compared to 10 mg kg^−1^ Se (VI). As presented in Table 1, SeMet could also be detected from the water-soluble fraction; although in much less amounts than Se (VI). In contrast to water-soluble fraction, the major detected peak of SAX HPLC-ICP-MS chromatograms was SeMet in the enzyme extract (Figure S1). The ratio of SeMet (major organic Se form) showed an increasing tendency during the consecutive harvests as a result of the decrease in the total Se content. Comparing the Se treatments, the relative ratio of SeMet form was the lowest in the 10 mg kg^−1^ Se (VI) treatment and the highest in 50 mg L^−1^ red Se^0^ treatment. However, it is worth noting that the ratio of SeMet accumulation in plants treated with 10 mg kg^−1^ Se (VI) (calculated as SeMet content in 10 mg kg^−1^ Se (VI)/SeMet content in 50 mg L^−1^ red Se^0^) was 0.4–3.6-fold greater than the 50 mg L^−1^ red Se^0^-treated plants. In contrast, the inorganic Se (VI) accumulation ratio (calculated by Se (VI) in 10 mg kg^−1^ Se (VI)/Se (VI) in 50 mg L^−1^ red Se^0^) was up to 55-fold higher in plants treated with 10 mg kg^−1^ Se (VI) than 50 mg L^−1^ red Se^0^ (Figure 2). In addition to the two major identified Se components, some minor unidentified selenocompounds were also detected (Figure S1).

### 2.2. Biochemical Changes in Alfalfa under the Exogenous Application of Different Se Forms

#### 2.2.1. Buffer-Soluble Protein

**Figure 3:**
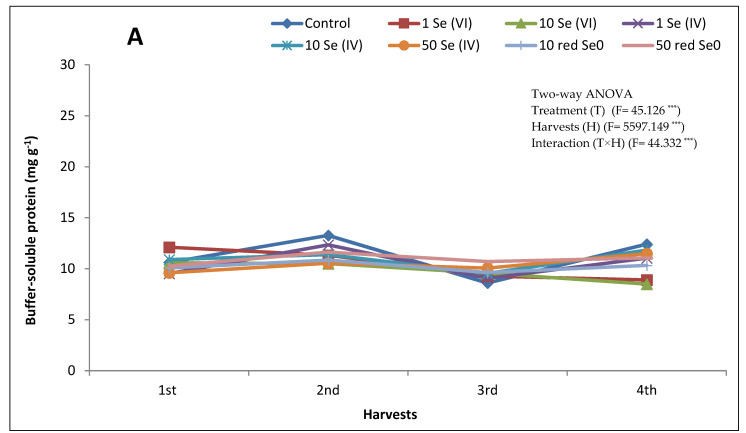

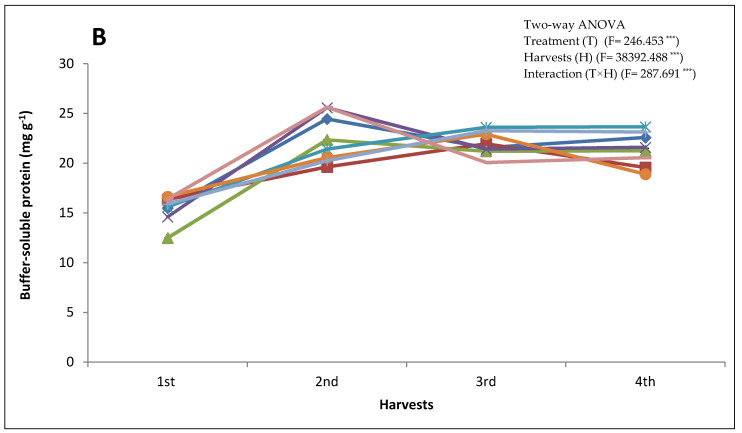
Buffer-soluble protein content in (**A**) stem and (**B**) leaves of alfalfa fertilized with different Se forms (i.e., Se (VI), Se (IV) and red Se^0^) at different concentrations (1, 10, and 50 mg kg^−1^ for ionic forms and 10 and 50 mg L^−1^ for red Se^0^) within four consecutive harvests with 30-day intervals. Alfalfa plants that received 50 mg kg^−1^ Se (VI) died two weeks after seed germination. Data are means ± SD (*n* = 6). *** significant according to Tukey’s test (*p* ≤ 0.001).

The content of buffer-soluble protein in alfalfa stem significantly varied upon the exogenous application of Se. Five treatments (i.e., control, 1, 10, and 50 mg kg^−1^ Se (IV) and 10 mg L^−1^ red Se^0^) out of the eight treatments exhibited the same response to the Se application regardless of its form and concentration within the four harvests; protein content decreased from the 1st harvest to the 2nd harvest followed by an increase in the 3rd harvest and then decreased in the 4th harvest. Although the Se (IV) treatments displayed the same trend of the protein content, no treatment resulted in the highest content of protein within the four harvests (Figure 3A). For instance, the highest protein content in the 1st, 2nd, 3rd, and 4th harvest (10.9, 12.3, 10.1, and 11.8 mg g^−1^, respectively) was 10, 1, 50, and 10 mg kg^−1^ Se (IV), respectively (Figure 3). Except for the 4th harvest, protein content in alfalfa stem grown in the presence of 50 mg L^−1^ red Se^0^ was higher than that of 10 mg L^−1^ red Se^0^. Moreover, the treatment of 50 mg L^−1^ red Se^0^ showed high protein content compared to the other Se treatments; even it was the highest in the 3rd harvest. Moreover, many Se treatments resulted in higher protein content in stem compared to control especially in the 1st and 3rd harvests. The 2nd harvest exhibited the highest protein content in stem followed by 1st, 4th, and 3rd harvest. Furthermore, within all harvests, the highest protein content belonged to the control followed by 10 mg kg^−1^ Se (IV), while the lowest content was measured when plants grew on 1 mg kg^−1^ Se (VI). On the other hand, protein content in leaves of alfalfa treated with Se displayed higher contents than control within the four harvests (Figure 3B). In general, the highest protein contents in the 1st and 2nd harvests were measured in alfalfa leaves treated with 50 mg L^−1^ red Se^0^. In contrast, the lowest protein contents in the 1st and 2nd harvests corresponded to the application of Se (VI), while in the 3rd and 4th harvests, the treatment of 50 mg L^−1^ red Se^0^ resulted in the lowest protein content. Moreover, application of red Se^0^ at the rate of 10 mg L^−1^ resulted in the highest protein contents 23.3 and 28.1 mg g^−1^ in the 3rd and 4th harvests, respectively. The treatment of 50 mg kg^−1^ Se (IV) exhibited a high protein content in the 1st and 3rd harvests. Among all harvests, the 2nd harvest was the best regarding the protein content followed by the 4th, 3^rd^, and 1st harvest. Regarding the treatments, the treatment of 10 mg L^−1^ red Se^0^ was the best followed by 10 mg kg^−1^ Se (IV).

#### 2.2.2. Lipid Peroxidation

The content of MDA in stem and leaves of alfalfa was measured as an indicator for the degree of oxidation of bilayer in the cell membrane. In the 1st harvest, the stem MDA content varied between 18.6 to 25.4 nmol g^−1^; however, the lowest value corresponded to the treatment of 10 mg L^−1^ red Se^0^, while the highest value was found in the treatment of 50 mg kg^−1^ Se (IV). Noticeably, addition of Se at concentration above 10 mg kg^−1^ resulted in high MDA content compared to the lower concentrations in the 1st harvest. Nevertheless, lower stem MDA contents were measured in the next harvests, 2nd to the 4th harvest, and ranged between 9.9 nmol g^−1^ (for 10 mg kg^−1^ Se (VI) in the 3rd harvest) and 13.4 nmol g^−1^ (for control in the 2nd harvest) as shown in Table 2. However, all Se treatments showed almost the same stem MDA content in the 2nd harvest where differences were insignificant. In the 3rd and 4th harvest, all treatments had stem MDA content similar to the control. No tendentious differences could be revealed between selenium treatments. With respect to the MDA content of the leaves, no big differences between harvests were measured as in the case of the stem. The MDA content of leaves changed between 14.7 and 21.2 nmol g^−1^ during the four consecutive harvests (Table 2). However, no tendentious differences were revealed between Se treatments, except the treatment of 50 mg L^−1^ red Se^0^ which exhibited the highest MDA content compared to all Se treatments. 

#### 2.2.3. Water-Soluble Phenols

In general, the content of water-soluble phenols was higher in alfalfa leaves than in stem regardless of the Se treatment and harvesting time. Most of the Se treatments caused an increase in the water-soluble phenol content of the stem which ranged from 36.8 to 77.3 µg g^−1^ (Table 2). For instance in the 1st harvest, the highest phenol content of stem was 67.5 µg g^−1^ in the presence of 10 mg kg^−1^ Se (VI), while control plants displayed the lowest phenol content 45.5 µg g^−1^. Furthermore in the 4th harvest, the highest phenol content changed between 72.8 and 77.3 µg g^−1^ using 50 mg kg^−1^ Se (VI) or 10–50 mg L^−1^ red Se^0^, while control plants exhibited 48.3 µg g^−1^. Regarding the Se treatments, the phenol content in leaves increased from the 1st to the 2nd harvest (the highest phenol content) then started to decrease gradually from the 3rd to the 4th harvest (lowest phenol content). Control plants had the lowest value (78 µg g^−1^) in leaves only in the 1st harvest. Phenol content shows a dose-response relationship to Se (VI) as it increased upon increasing the Se concentration from 1 to 10 mg kg^−1^. However, similar responses did not report for the Se (VI) treatments, which showed hesitating phenol contents. The red Se^0^ displayed the same response as Se (VI) where phenol content was higher when red Se^0^ was applied at the rate of 50 mg L^−1^ compared to 10 mg L^−1^. Regardless of the harvests, the application of Se (VI) at the rate of 10 mg kg^−1^ recorded the highest phenol content in leaves. All Se treatments resulted in lower phenol contents in 2nd, 3rd, and 4th harvests compared to control with slight exceptions. 

#### 2.2.4. Peroxidase Activity

The activity of peroxidase enzyme (POD) showed the same response in the stem and leaves of alfalfa. In general, the high concentrations of the applied Se resulted in lower levels of POD activity, while the low concentrations induced the POD activity compared to the control in all the harvests with slight exceptions. For instance, stem POD activity increased from 12.2 to 45.3 U mL^−1^ min^−1^ g^−1^ DW when Se (VI) was applied at the rate of 10 and 1 mg kg^−1^, respectively (Table 2). Similar findings were reported in the 3rd harvest for the same treatments where 1 mg kg^−1^ Se (VI) recorded higher POD activity than 10 mg kg^−1^ Se (VI) recording an increase of 442.7%. Applying Se at the rate of 1 mg kg^−1^ in the form of Se (VI) and Se (IV) recorded the highest activity of POD within all harvests. Likewise, in alfalfa leaves, the treatment of 10 mg kg^−1^ Se (VI) resulted in the lowest POD activity compared to the other Se treatments including the control. Moreover, 1 mg kg^−1^ Se (VI) had higher POD activity than 10 mg kg^−1^ Se (VI) in all harvests except the 1st harvest. The addition of red Se^0^ at the rate of 10 mg L^−1^ recorded an increase in POD activity compared to 50 mg L^−1^ in stem and leaves. 

### 2.3. Plant Biometrics

#### 2.3.1. Shoot Length

Length of the aboveground part of alfalfa (shoot) significantly responded to the exogenous application of Se. Moreover, shoot length shows a negative dose-response relationship to the Se application as high Se concentrations, i.e., 10 mg kg^−1^ Se (VI) and 50 mg kg^−1^ Se (IV), significantly diminished the shoot length in all harvests compared to low Se concentrations (Table 3). Application of Se at the rate of 1 mg kg^−1^ as Se (VI) resulted in the tallest shoot compared to other Se concentrations and forms; however, 10 mg kg^−1^ Se (VI) resulted in the shortest shoot in the 1st and 2nd harvests. However, in the 3rd and 4th harvest 10 mg kg^−1^ Se (VI) had less negative impact of shoot length as an increase in shoot length was measured compared to the 1st and 2nd harvests. On the other hand, Se (IV) showed a different effect as the 10 mg kg^−1^ treatment exhibited a taller shoot than 1 and 50 mg kg^−1^ treatments. In general, plants grew on 1 mg kg^−1^ Se (VI), 10 mg kg^−1^ Se (IV), and 50 mg L^−1^ red Se^0^ displayed the highest shoot length within the four harvests. Among the four harvests, the 3rd harvest possessed the tallest shoot followed by the 4th harvest while the shortest shoot was measured in the 1st harvest.

#### 2.3.2. Shoot Dry Mass

Treatments with high Se concentrations, i.e., 10 and 50 mg kg^−1^ of Se (VI) and Se (IV), respectively, had the lowest dry mass of shoot in all harvests; however, in the 3rd harvest, they recorded higher dry masses. Control plants had the highest dry mass only in the 1st harvest; while, Se treatments resulted in higher dry masses in the other harvests (Table 3). Treatments with low Se concentrations, i.e., 1 mg kg^−1^ Se (VI), 10 mg kg^−1^ Se (IV), and 50 mg L^−1^ red Se^0^, showed higher shoot dry masses. For example, in the 2nd harvest, the shoot dry mass was 1.0 g plant^−1^ at 1 mg kg^−1^ Se (VI) and lowered to 0.4 g plant^−1^ when Se (VI) concentration increased to 10 mg kg^−1^. Likewise, 10 mg kg^−1^ Se (IV) had a better effect on shoot dry mass compared to 50 mg kg^−1^ as higher dry masses were measured. Contrary to the two ionic forms of Se (Se (VI) and Se (IV)), red Se^0^ exhibited an opposite effect on the shoot dry mass as 50 mg L^−1^ had higher dry masses than 10 mg L^−1^. Among harvests, the 3rd harvest displayed the highest shoot dry mass followed by the 2nd harvest. 

### 2.4. Pearson Correlation and PCA Analysis

**Figure 4:**
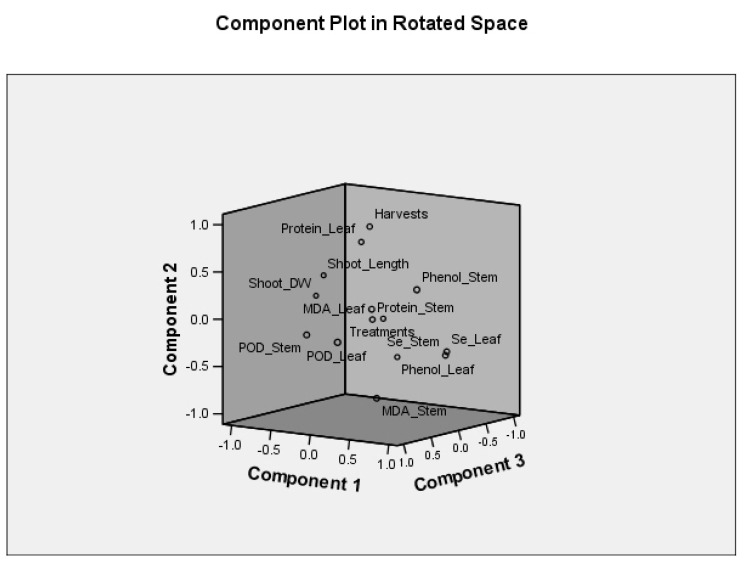
Principal component analysis of the morphological, chemical and biochemical properties of alfalfa fertilized with different Se forms (i.e., Se (VI), Se (IV), and red Se^0^) at different concentrations (1, 10, and 50 mg kg^−1^ for ionic forms and 10 and 50 mg L^−1^ for red Se^0^). Data are means (*n* = 6).

Running the principal component analysis (PCA) using SPSS 13.0 generated 14 components (PC) in which the first 5 PCs explained 80.2% of the total variance (Figure 4 and Table S3). The PC-1 to PC-5 explained 31.3, 17.5, 14.7, 8.9, and 7.7%, respectively. The PC-1 basically described the total Se content in stem and leaf, MDA content in stem, protein content in leaf, shoot length, and shoot dry mass (Table 4). On the other hand, leaf MDA content and phenol content in stem and leaf of alfalfa were designated by the PC-2. While PC-3 largely defined the activity of POD in both stem and leaf, protein content in stem was labeled to the PC-4. Harvest time and Se treatments were explained by the PC-5. 

Positive and negative correlations between the measured parameters of alfalfa were reported by Pearson correlation (2-tailed). For instance, harvests displayed a moderate, negative and significant correlation with total Se content in stem (*p* < 0.05) and phenol content in leaf (*p* < 0.05) and high, negative, and significant correlation with MDA content in stem (*p* < 0.01). Harvests, also, showed a moderate, positive and significant correlation with protein content in leaf (*p* < 0.01) and shoot length (*p* < 0.05). The content of MDA in leaf was the only character that showed a significant positive correlation with the Se treatments (Table S2). On the other hand, total Se content in stem significantly correlated with MDA content in stem (*p* < 0.01; moderate and positive), leaf protein content (*p* < 0.05; moderate and negative), shoot length (*p* < 0.01; moderate and negative), and shoot dry mass (*p* < 0.01; moderate and negative). The same correlations were reported for the total Se content in leaf. All of leaf protein content (*p* < 0.01), shoot length (*p* < 0.01) and shoot dry mass (*p* < 0.05) displayed significant and negative correlation with the MDA content in stem. Contrarily, MDA content in stem showed a positive and significant correlation with POD activity in leaf (*p* < 0.01). Leaf MDA content resulted in positive and significant correlations with stem phenol content (*p* < 0.01) and POD activity in leaf (*p* < 0.05) and negative correlation with shoot dry mass (*p* < 0.05). Both leaf protein content and shoot dry mass showed positive and significant correlations with shoot length. A high positive and significant correlation (*p* < 0.01) between shoot length and shoot dry mass was reported. 

## 3. Discussion

Several plant crops and forages lack Se due to low soil Se and/or poor Se-accessibility for plant uptake where many factors affect Se-phytoavailability such as soil pH, redox potential (Eh), organic matter, soil texture, total soil Se content, and the chemical form of Se [9,11,29,30]. Consequently, many techniques have been suggested to elevate Se content in food and feed to fulfill the daily Se requirements for humans and animals [9]. Yet, the agronomic biofortification of Se, added as either soil or foliar application, has recently gained extraordinary attention due to its easy, safe, and effective application. Treating growing plants/forages by different chemical Se forms not only increases the total Se content in plant biomass but also alerts the content of several bioactive compounds [9,31]. 

In the present study, we did not only aim to investigate the efficacy of the Se-enrichment process of alfalfa (“king of forages”) but also to study the effect of different chemical Se forms (i.e., Se (VI), Se (IV), and red Se^0^), concentrations, and Se-uptake dynamics within four consecutive harvests on the total Se content; Se forms in alfalfa aboveground biomass were also examined alongside the changes in some chemical, biochemical, morphological parameters. There were two reasons beyond selecting alfalfa as a role model. First reason is that alfalfa is a multi-harvested plant, and thus it is a good model to compare the uptake and translocation dynamics of different Se forms in newly regrown leafy shoots. Second reason is that alfalfa has a great importance as an alternative source of protein alongside soybean in the continental climate zone. Beyond traditional uses such as hay production, these days alfalfa is considered as a promising crop in the novel green biorefinery concept [32,33]. Green biorefinery processes convert the raw green biomass into a range of marketable products including feed, food, chemicals, biofuels, heat (multi-product-system) based on deployment of sustainable zero-waste technologies. By developing a well-managed forage fortification method, organic Se-enriched products can be produced from the yearly four–six times harvested and fractionated alfalfa green biomass. Although several studies have been published in the topic of alfalfa selenium fortification [34], according to our knowledge, the present work is the first in which the Se uptake and transportation conversion dynamics of three different inorganic forms are compared during several consecutive harvests, covering the entire growing season.

Selenium is one of the essential microelements known for its double-edged action as it exhibits indispensable benefits when applying at low concentrations while the high concentrations are lethal. This was confirmed by our results where plants exposed to 50 mg kg^−1^ Se (VI) were not able to survive after germination. The toxicity of high Se doses is mainly attributed to the incorporation of Se-amino acids into proteins that disrupts protein folding and thus causing loss of functions [35]. However, this depends heavily on plant Se/S ratio rather than Se content alone. Furthermore, the high accumulation of Se in plant tissues causes developmental malformations, and induces the overexpression of reactive oxygen species (ROS) due to reaction of Se with the thiol group and GSH [36], and stimulates protein nitration, especially tyrosine nitrate [37]. However, methylation of Se-amino acids can alleviate their toxicity, where mainly SeCys and SeMet are converted into methyl-SeCys and methyl-SeMet, respectively, and turned into volatile Se forms, i.e., dimethyl selenide and dimethyl diselenide [38]. Along with it, the selenium tolerance and accumulation ability of hyperaccumulator or primary accumulator plant species are due to diverse metabolic pathways. The accumulation of selenohomocysteine (SeHCy) derivatives related to fatty acid metabolism and also polyselenides can be detected from some plant species [39].

Our results also revealed that the uptake, translocation, and accumulation of Se in alfalfa tissues heavily depend on chemical form and applied concentration of Se as well as the harvesting time. The chemical form of Se in the active root zone largely affects its uptake. For instance, Se (VI) is the most mobile, soluble, and absorbed ionic form, especially in oxic soil (aerated) where Se is found in its highest oxidized state (6^+^); and consequently, it poorly adheres to soil complexes and oxides [28]. Plant roots uptake Se (VI) through sulfate transporters, i.e., SULTR 1 and 2, and Se (VI) is actively transported through the cell membrane [40]. Our findings are in the same line as the highest total Se content in stem and leaf of alfalfa, especially in the 1st and 2nd harvests, corresponded to the application of Se (VI) at the rate of 10 mg kg^−1^. However, total Se content in stem and leaf of plants fertilized with Se (VI) linearly and significantly decreased within the harvests. For instance, in the treatment of 10 mg kg^−1^ Se (VI), the total stem Se content was 202.5 µg g^−1^ in the 1st harvest and dropped down to 14.6 µg g^−1^ in the 4th harvest (Figure 1). Similar results were reported in leaf for the same treatment, with higher Se content than the stem, as in the 1st harvest, the total Se content was 643.4 µg g^−1^ and declined to 58.1 µg g^−1^ in the 4th harvest (Figure 1). Two reasons could be behind this high rate of Se (VI) uptake in the first two harvests and the low rate in the next consecutive harvests, i.e., 3rd and 4th harvests: (1) the short root system of alfalfa in the first harvests compared to late harvests as the upper layer of soil is more aerated than the deeper one that ensure the existence of Se in form of Se (VI) due to the oxic conditions [33], and (2) the high dose of applied Se (VI), i.e., 10 mg kg^−1^. However, this cannot be referred to the low sulfur soil content since Se (VI) is absorbed through the same transporters as sulfate because the sulfur content in the experimental soil was 220 ± 5.6 mg kg^−1^ (Table S3) which is much higher than the recommended sulfur soil content 10–20 mg kg^−1^ [34].

On the other hand, in soil that lacks aeration (anoxic), Se (IV), the less mobile and available Se from, is the dominant form, while further reduction of Se (VI) can lead to selenide (2-) and Se^0^ forms [33]. In our experiment, alfalfa root absorbed Se (IV) at a lower rate than Se (VI); 1 mg kg^−1^ Se (VI) resulted in total stem Se content higher than that of 10 mg kg^−1^ Se (IV) in the 1st harvest. Nevertheless, in the successive harvests, 10 mg kg^−1^ Se (IV) exhibited slightly higher total Se content in the stem. Regarding total Se content in leaf, 10 mg kg^−1^ Se (IV) displayed higher content than 1 mg kg^−1^ Se (VI) in all harvests (Figure 1). Interestingly, the treatment of 50 mg kg^−1^ Se (IV) showed the second-highest total Se content in stem and leaf of the 1st and 2nd harvests, while it resulted in the highest total Se content in the 3rd and 4th harvests. 

Currently, it is well-known that the Se (IV) is mainly transported through the cell membrane by the phosphorus and silicon transporters, i.e., NIP2 and 1 and PHT2 [32]. Therefore, in soils rich in phosphorus—such as our experimental soil (2015 ± 135 mg kg^−1^; Table S3)—Se (IV) competes with phosphorus for NIP2;1 and PHT2 transporters. Consequently, the low uptake rate of Se (IV) by alfalfa root in the present experiment could be attributed to the suppression of Se (IV) uptake by high soil phosphorus content, particularly for the first two harvests. Moreover, the high soil organic matter content (18.69 ± 0.14 g kg^−1^; Table S3) could be a reason behind the low rate of Se (IV) uptake due to the retention of Se (IV) on the organic matter [16]. 

Recently, there is an emerging attitude to use the Se^0^ for Se biofortification of plant crops rather than the ionic forms, i.e., Se (VI) and Se (IV) [9]. Our results showed promising findings related to the utilization of red Se^0^ in biofortification of crops/forages by Se. The application of red Se^0^, particularly at the rate of 10 mg L^−1^, showed the same effect as slow-release fertilizers, as the accumulation of total Se in the stem and leaf of alfalfa during the successive harvests remained almost the same, contrary to what the Se (VI) and Se (IV) displayed. The treatment of 50 mg L^−1^ red Se^0^ also showed promising traits. Total stem Se content after treating alfalfa by 10 mg L^−1^ red Se^0^ was 6.9, 8.3, 6.0, and 10.5 µg g^−1^ in the 1st, 2nd, 3rd, and 4th harvest, respectively (Figure 1). Nevertheless, the same treatment showed the same effect but with a gradual increase in the total leaf Se content within the harvests recording 14.3 µg g^−1^ in the 1st harvest and increased to 37.5 µg g^−1^ in the 4th harvest (Figure 1). Various factors affecting the uptake of nanoparticles like red Se^0^ such as physical and chemical properties of nanoparticles, plant type, growth medium, and/or plant-soil-microbe interactions [33]. Some nanoparticles can form complexes with organic compounds secreted from plant roots and/or soil microorganisms. There are two proposed pathways for the uptake of nanoparticles; however, this depends on the particle size of nanoparticles. The apoplastic route is suggested to be the path of small nanoparticles below 20 nm as it can easily penetrate the cell membrane of root tissues [34]. On the other hand, larger nanoparticles can infiltrate the plant root via the symplastic route through the inner side of the plasma membrane [34]. However, red Se^0^ could find its way into root cells first through its oxidation in soil by some bacteria, i.e., *Bacillus megaterium*, into Se (IV) and consequently taken up via the phosphorus transporters or even could be further oxidized into Se (VI) and pass through sulfate transporters [35]. 

Broadley et al. [41] cited that plants can uptake approximately 12% of Se added as soil application as the main portion of Se becomes unavailable due to the fixation on soil particles and organic matter. Consequently, they recommended the foliar application of Se fertilizers for better agronomic biofortification. However, opposite findings were reported by da Silva et al. [42]; they investigated the uptake of two Se forms, i.e., sodium selenate and sodium selenite, applied at the rate of 1.2 mg kg^−1^ as a soil application and 50 µmol L^−1^ as a foliar application by radish (*Raphanus sativus* L). Soil application of sodium selenate resulted in higher Se content in the root, leaf of radish compared to foliar application of the same form. Moreover, soil application of sodium selenite showed the same response but to a lower extent. Thus, they recommended soil application of selenate as the best technique for Se biofortification. Our results showed that 10.2% of applied Se at the rate of 10 mg kg^−1^ Se (VI) was taken up by alfalfa plants on average. On the other hand, 6.1% and 4.9% of applied 10 mg L^−1^ red Se^0^ were taken up by plants in the 3rd and 4th harvests, respectively. Considering the uptake dynamics and variation in Se uptake and accumulation in alfalfa tissues, it could be concluded that 10 mg L^−1^ red Se^0^ is the best treatment from the Se-biofortification point of view as it displayed almost a constant uptake rate of Se. As a total of the four harvests, the highest Se uptake % was 18.4% for 10 mg kg^−1^ Se (VI) followed by 17.2% for 10 mg L^−1^ red Se^0^. 

Plants can absorb inorganic and organic Se forms from soil solution; however, there is a higher affinity for organic Se compounds such as Se-amino acids [10]. Directly after entering the root cells, ionic Se forms, Se (VI) and Se (IV) are transported to plastids to subsequently synthesize Se-amino acids, i.e., SeCys and SeMet [43]. The absorbed Se (VI) has to be reduced to Se (IV) before the synthesis of SeCys; however, the reduction of Se (VI) is energy-consuming and thus is a limiting step that is regulated by the ATP sulfurylase enzyme [44]. 

The results of the separation of different chemical Se forms in alfalfa tissues HPLC-ICP-MS showed that the Se (VI) and SeMet were the dominant Se forms with few peaks for unknown Se forms. The Se (VI) content in stem or leaf shows a high dependence on applied Se form, concentration, and harvesting time. The highest Se (VI) content corresponded to the treatment of 10 mg kg^−1^ Se (VI) followed by 50 mg kg^−1^ Se (IV), while the lowest measured Se (VI) content was for the application of red Se^0^ at the rate of 10 mg L^−1^ (Table 1). In general, the leaf possessed higher Se (VI) and SeMet content compared to the stem. The high total Se content in the leaf may help explain this phenomenon, in addition to the fact that the synthesis of organic Se compounds mainly occurs in plastids [44]. Consuming organic Se forms by humans/animals is better than the ionic Se supplements especially for antioxidant enzymes [35]. Most enzymes, especially the antioxidant ones, work on organic Se forms better than the ionic ones; so, it has to be aimed not only at elevating the total Se content in crops/forages but also increasing the content of the organic Se compounds such as SeMet and SeCys. SeMet is known as the major organic Se form in Se-non-accumulators such as alfalfa [36,37,43]. The SeMet/total Se ratio ranged between 10.0% and 63.3% in the alfalfa stem and leaves depending on the applied Se treatment and harvesting time (Figure 2A,B). The treatment of 10 mg kg^−1^ Se (VI) resulted in the highest SeMet (water extract) only in the 1st harvest; though, the highest SeMet content in the 2nd and 4th harvests was for the application of Se (IV) at the rate of 50 mg kg^−1^. Moreover, measuring Se (VI) and SeMet in enzymatic extract displayed a much higher concentration than water extract (Table 1). Considering the applied Se concentration, treatment of 50 mg L^−1^ red Se^0^ exhibited higher SeMet yield %. Interestingly, the recovery percentage of SeMet in all the treatments is largely affected by the harvesting time. The highest SeMet yield % was measured in the 4th harvest followed by the 2nd one, whereas the lowest recovery percentage was detected in the biomass of the 2nd harvest. In stem, the SeMet yield %, after the treatment of 50 mg L^−1^ red Se^0^, was 28.6% in the 1st harvest then increased to 63.6% in the 4th harvest. Likewise, in the leaf, 25.7% and 38.0% as SeMet yield % were calculated in the 1st and 4th harvests, respectively, in plants treated with 50 mg L^−1^ red Se^0^ (Figure 2). Dong et al. [38] investigated the different Se forms in potato tubers after treating the plants with 200 µg mL^−1^ sodium selenite, added as a foliar application. They cited that in the gastrointestinal digested raw potato tubers, no Se (VI) was detected while other organic forms were measured; and they were in the following order: Se (IV) > SeMet > SeCys > SeMeCys (Selenomethylcysteine). Yet, they measured very low concentrations of these organic Se forms compared to our experiment. However, this could be attributed to plant species, applied Se concentration, and the application method of Se. Finally, it cannot be overlooked that the biofortification of Se using red Se^0^ form is the building of Se into organic form (especially SeMet), which is more needed and important for consumption by humans and animals.

Application of Se to plants with the purpose to elevate Se content in plant tissues might have additional benefits for plant growth; Se can directly or indirectly serve as an antioxidant, especially when applied at low concentration [42]. Malik et al. [43] reported a decline in electrolytic leakage upon the repair of cell membrane fluidity by Se under different abiotic stresses. Moreover, improvement in scavenging oxidants, generated in plant tissues under various stress circumstances, has been documented upon the application of Se which enhances the activity of several antioxidant enzymes including POD, GSH, catalase (CAT), superoxide dismutase (SOD), and ascorbate peroxidase (APX) [44]. Consequently, low Se concentration reduces the level of ROS, leading to a delay in plant senescence [44,45]. Moreover, low Se dose has been cited to improve plant biometrics, e.g., photosynthetic pigments, relative water content, and osmolytes (soluble protein, proline, sugars, and ions), particularly under salinity stress [39] and waster deficit [40]. Leaves of alfalfa exhibited higher content of buffer-soluble protein compared to the stem. The 1st harvest showed the lowest buffer-soluble protein content in the leaf, while in the stem the lowest content belonged to the 3rd harvest. The treatment of 10 mg kg^−1^ Se (VI) showed the lowest buffer-soluble protein content in the stem, whereas red Se^0^ added at the rate of 50 mg L^−1^ resulted in the lowest leaf buffer-soluble protein content (Figure 3). Similar findings were reported for MDA and water-soluble phenol contents as the highest applied Se concentration, regardless of its chemical form, possessed the highest content. Moreover, the treatment of 10 mg kg^−1^ Se (VI) showed the lowest POD activity in stem and leaf in all the harvests, except 1st harvest leaf. This could be referred to mishandling or measuring error. 

Many studies have reported an increase or no variations in plant biomass upon treating plants with different chemical forms of Se. In the present study, however, low Se concentrations exhibited the same effect on plant biomass, while a reduction in length and dry mass of alfalfa shoot has been noticed for treatments of 10 mg kg^−1^ Se (VI) and 50 mg kg^−1^ Se (IV) compared to other Se treatments and control, particularly in the first two harvests (Table 3). Furthermore, lower MDA content and balanced Na^+^ homeostasis have been also noticed for plants fertilized with low Se concentrations. On the other hand, high Se concentrations are detrimental to plant growth. It causes developmental malformations, and induces the overexpression of ROS due to reaction of Se with the thiol group and GSH [36,46], and stimulates protein nitration, especially tyrosine nitrate [37]. Our results are consistent with these findings where the total stem Se content measured after the treatments of 10 mg kg^−1^ Se (VI) and 50 mg kg^−1^ Se (IV), the highest two applied Se doses, was 202.5 and 145.3 µg g^−1^, respectively, in the 1st harvest and decreased to 98.0 and 71.7 µg g^−1^, respectively, in the 2nd harvest. Moreover, in leaf, total Se in the 1st harvest was 643.4 and 390.5 µg g^−1^ for treatments of 10 mg kg^−1^ Se (VI) and 50 mg kg^−1^ Se (IV), respectively, whereas in the 2nd harvest it was 284.5 and 215.1 µg g^−1^, respectively (Figure 1). Furthermore, results of Se speciation of stem and leaf of alfalfa in the 1st, 2nd, and 4th harvests, to a large extent, support our conclusion regarding the hazardous impact of high Se doses on plant growth and development. The 10 mg kg^−1^ Se (VI) and 50 mg kg^−1^ Se (IV) treatments, the highest Se forms accumulated in the stem is the Se (VI) recording 79.5 and 89.0 µg g^−1^, respectively, in the 1st harvest, while 50 mg kg^−1^ red Se^0^ treatment resulted in Se (VI) content of 6.47 µg g^−1^ (Table 1). The content of Se (VI) in the stem of the 2nd harvest was lower than that of the 1st harvest but it was higher than those of the 4th harvest. Higher Se (VI) content was measured in alfalfa leaf within the 1st, 2nd, and 4th harvests compared to stem. For instance, 901 and 336 µg g^−1^ of Se (VI) was determined in the leaf of plants treated with 10 mg kg^−1^ Se (VI) and 50 mg kg^−1^ Se (IV), respectively. On the contrary, very low Se (VI) content was measured in stem and leaf of alfalfa plants grown on 50 mg L^−1^ red Se^0^ in all the harvests compared to the addition of the ionic Se forms, Se (VI) and Se (IV). 

Finally, it could be concluded that the application of the Se fertilizers to growing plants not only achieved an increase of total Se content in food/feed but also enhanced the nutritional quality of food/feed by increasing the content of several phytochemicals with health benefits to humans and animals [32]. Selenoproteins are considered as direct antioxidants since they can be easily oxidized. The precursors of selenoproteins in mammalian cells are selenocysteine (SeCy) and SeMet [9]. Moreover, several organic-Se compounds such as SeMet were identified as anti-cancer agents [47]. The present study on red Se^0^ afforded promising findings that should be intensively investigated in the near future. Alongside its success to produce Se-enriched alfalfa, it also showed the highest recovery percentage of SeMet, which is more biologically important than ionic Se forms, i.e., Se (VI) and Se (IV). Our experiments demonstrated that red Se^0^ has the same behavior as a slow-release fertilizer, from the agronomy point of view is important to ensure a continuous supply of Se to produce biomass with almost a constant Se content. Another important finding is that the leaves of alfalfa are richer in Se and buffer-soluble protein compared to stem; consequently, losing alfalfa leaves during its drying to convert fresh biomass into hay or even during the harvest is considered a big loss. Therefore, isolation of leaf protein from fresh alfalfa biomass could be an effective alternative solution to introduce Se-fortified and protein-rich biomass to animals. 

## 4. Materials and Methods

### 4.1. Plant Source and Soil Materials

Seeds of alfalfa (*Medicago sativa* L. var. Tápiószelei 1) were kindly provided by Tedej ltd, Hungary. Soil used in the present study was top-soil (0–25 cm) and collected from the Experimental Farm of University of Debrecen, Debrecen, Hungary (47°32′0″ N; 21°38′0″ E). Air-dried soil samples were partially crushed and sieved through 4 mm sieve and used for growing alfalfa plants. Physicochemical properties of soil are presented in Table S3 (supplementary materials).

### 4.2. Chemical Synthesis of Red Elemental Se 

Red elemental Se (red Se^0^) in its nano-size was prepared using ascorbic acid as a reducing agent for sodium selenite in the presence of sodium dodecyl sulfate (SDS) as a stabilizer. Briefly, 250 mL of 50 mM L-ascorbic acid (C_6_H_8_O_6_-99%, Merck-Sigma, Darmstadt, Germany) was added dropwise to 500 mL of 12.7 mM sodium selenite (Na_2_SeO_3_-99%, Merck-Sigma, Darmstadt, Germany) with vigorous stirring. Afterward, 1 g SDS (C_12_H_25_NaO_4_S, Merck-Sigma, Darmstadt, Germany) was added to the solution to prevent the aggregation of the produced particles of red Se^0^. The solution was stirred continuously for 3 h until its color turned to red as an indication of formation of the red Se^0^ particles. After that, Milli-Q water (Millipore, Molsheim, France) was added to bring the total volume of solution to 1 L. All solutions were prepared using Milli-Q water. Solution was centrifuged at 10,000 rpm at room temperature to collect the red Se^0^ particles. The pellets were re-suspended in Milli-Q water and dispersed by sonication (Sonics VCX 750, Sonics & Materials Inc., Newtown, CT, USA) before measuring the final concentration of Se using inductively coupled plasma optical emission spectroscopy (ICP-OES, model Vista-Pro from Varian) and preparing the treatments. 

### 4.3. Characterization of Red Se^0^

**Figure 5:**
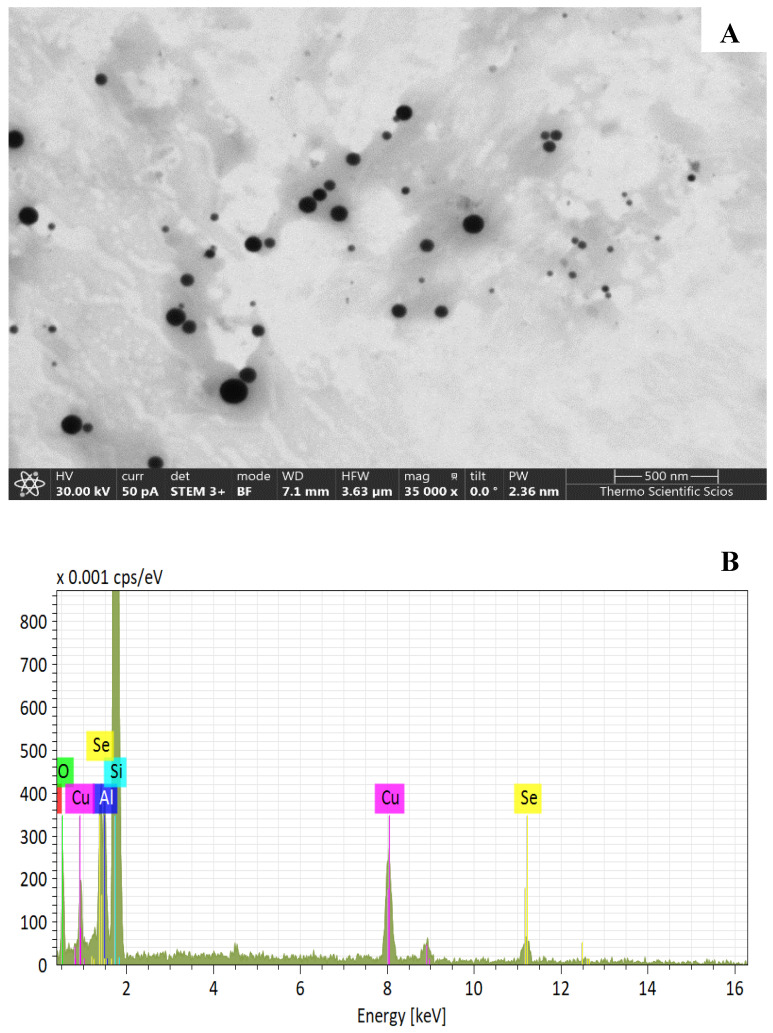
(**A**) The shape and size of chemically synthetized red Se^0^; (**B**) elemental composition of red elemental particles.

Particle shape and size of chemically synthetized red elemental selenium were examined by Thermo Scientific Scios 2 DualBeam Scanning Electron Microscope (Waltham, MA USA). It was operated at 30.0 keV accelerating voltage in STEM mode using the STEM 3+ transmission detector. During sample preparation, the red Se^0^ sol was applied on the amorphous carbon-precoated copper grid (d = 3 mm). The size of regular spherical elemental selenium particles varied in the range of 60 to 150 nm (Figure 5A). The elemental composition of the Se-containing sol was also observed by the SEM technique. The other elements next to Se came from the sample holder (Figure 5B).

### 4.4. Experimental Setup

The experimental layout was the randomized complete block design (RCBD) with seven repetitions. Alfalfa seeds were sown in plastic pots (8 kg air-dried soil, no holes) in a greenhouse environment at a rate of 0.29 g seed per pot. Three different chemical forms of Se were applied, such as sodium selenate (Se (VI)), sodium selenite (Se (IV)), and red Se^0^, in addition to a control which received no Se. Both Se (VI) and Se (IV) were applied at three different concentrations, i.e., 1, 10, and 50 mg kg^−1^ prepared from sodium selenate (Na_2_SeO_4_) and sodium selenite Na_2_SeO_3_), respectively, while red Se^0^ was applied at two different rates, i.e., 10 and 50 mg L^−1^. Addition of Se forms was carried out before seed sowing in 1 L water to stay safely below the water holding capacity (WHC) of used soil (WHC was 360 mL H_2_O kg^−1^ soil at saturation). For red Se^0^ treatments, each pot received 1 L of each concentration. Pots were irrigated by tap water and kept at no higher than 75% water saturation during the entire period of experiment (checked by weighing the pot each time it was watered). The first harvest was carried out 39 days after seed sowing while the following harvests were done in about 30-day intervals. Considering the alfalfa harvest practice, it was done at the green flower stage. 

### 4.5. Selenium Measurement

After harvest, shoot part was washed thoroughly with deionized water to remove any adhered Se to plant surface. After air drying, shoots were separated into stem and leaves, and then lyophilized using an Alpha 1–4 LSC Christ lyophilizer (Martin Christ GmbH, Germany). After lyophilization, 200 mg sample was placed in a Kjeldahl digestion tube and digested with 3 mL HNO_3_ at 120 °C for 60 min in block digestion unit. After cooling off, 2 mL H_2_O_2_ was added to the digestion tube then heated again to 150 °C for an additional 60 min. To complete the reduction of selenate into selenite, 20 mL 6 M HCl was added to cooled sample and heated to 100 °C for 60 min. The total volume of digested sample was brought up to 50 mL using Milli-Q water. The total Se content in the atomized hydride was determined via atomic fluorescence spectrometry (AFS) technique with PS Analytical Millenium machine (PS Analytical Ltd., Orpington, England). For measuring, Se hydride was generated in a flow injection system. A flow rate of 1.5 mL min^−1^ (3 M hydrochloric acid) and a similar flow rate of the reductant solution (1.4 m/v % sodium tetrahydroborate) were used to generate the Se hydride.

### 4.6. Determination of Chemical Forms of Se in Alfalfa

#### Sample Preparation

For water extraction, ~200 mg lyophilized sample was mixed with 9 mL Milli-Q water and then incubated in ultrasonic bath for 10 min at room temperature. The sample was centrifuged at 9000× *g* for 10 min. The supernatant was collected in new Falcon tube and dithiothreitol (DTT) (Merck-Sigma, Darmstadt, Germany) was added to reach 0.01% final concentration DTT in the sample. The water-extracted sample was filtered through 0.22 µm hydrophilic PTFE disposable syringe filters. The pellet was kept for enzymatic preparation.

For enzymatic extraction, 40 mg protease XIV enzyme (Merck-Sigma, Darmstadt, Germany) was dissolved in 9 mL 100 mM TRIS buffer (pH 8) and added to the pellet collected during the preparation of water extracts. It was shaken at 37 °C for overnight. After the adjustment of pH, an additional 40 mg of protease XIV dissolved in 1 mL TRIS was added to the sample. It was shaken again at 37 °C for 6 h. The sample was centrifuged at 9000× *g* for 10 min at room temperature. The supernatant was collected in Falcon tube and dithiothreitol was added to reach 0.01% final concentration DTT in sample. The enzymatic-extracted sample was filtered through 0.22 µm hydrophilic PTFE disposable syringe filters. Enzymatic extraction was also performed with SELM-1 as a reference material (LGC Standards GmbH, Germany) in parallel with the samples.

### 4.7. Anion Exchange Chromatography (SAX)

The separation of chemical Se forms was conducted by a high-performance liquid chromatography coupled with a plasma mass spectrometry system (HPLC-ICP-MS). The chromatographic set-up consisted of a Thermo Spectra System P4000 HPLC pump (Thermo Fisher Scientific, Waltham, MA, USA) connected to the Thermo Scientific X-Series II ICP-MS for element-specific detection of ^78^Se and ^80^Se, using 7% H_2_-93% He as collision gas, introduced at 6.0 mL min^−1^. A PRP-X100 SAX column (250 mm × 4.1 mm × 10 µm; Hamilton, Reno, NV, USA) was used with 100 µL injection volume. Gradient elution was performed with ammonium-acetate (buffer A: 10 mM; buffer B: 300 mM, pH 5.5) delivered at 1.9 mL min^−1^. The program was 0 min 0% B; 0–8.5 min 85% B; 8.5–8.6 min 0% B; 8.6–11.5 min 0% B.

The SeMet yield % was calculated to evaluate the efficiency of biotransformation of inorganic Se forms into SeMet. The SeMet yield % was calculated using the following formula:

$$\mathrm{eMet}\;\mathrm{yield}\;\%=\frac{\left[\mathrm{SeMet}\;\mathrm{in}\;\mathrm{water}\;\mathrm{extract}\right]+\left[\mathrm{SeMet}\;\mathrm{in}\;\mathrm{enzyme}\;\mathrm{extract}\right]}{\left[\mathrm{Soil}-\mathrm{applied}\;\mathrm{Se}\right]}*100$$

### 4.8. Plant Biometrics and Biochemical Analyses 

At every harvest, shoot length, fresh and dry masses of stem and leaves were determined. Soluble protein in stem and leaf tissues was determined using Coomassie Brilliant Blue G-250 according to Bradford [48] in triplicate with bovine serum albumin as standard with slight modifications. Briefly, 20 mg lyophilized plant tissue was pulverized in the mortar with quartz sand in the presence of 1 mL buffer (for 100 mL buffer: for 100 mL buffer: 0.6 g NaCl, 21 g urea, 6.6 g thiourea and 0.4 g NaOH), then transferred into a 1.5 mL Eppendorf tube. The homogenate was centrifuged at 10.000 rpm for 3 min. The supernatant was used for the assay of soluble protein using UV-160A spectrophotometer (Shimadzu, Japan) at 595 nm.

Peroxidase (POD) activity in alfalfa stem and leaves was determined according to Roxas et al. [49]. Briefly, 200 mg lyophilized sample was powdered in 1 mL phosphate buffer 250 mM (pH 6.8). The homogenate was centrifuged at 13,000 rpm for 10 min to collect the supernatant. The supernatant was used to measure GPX activity using UV-160A spectrophotometer (Shimadzu, Japan) at 460 nm for 1 min. The activity of GPX was expressed as unit 1 mL^−1^ 1 min^−1^ 1 g^−1^ dry mass. The unit of GPX activity was defined as the increase of one unit of absorbance per mL in 1 min for 1 g of dry matter. 

Malondialdehyde (MDA) content in stem and leaves of alfalfa as an indicator of degree of lipid peroxidation was determined as described by Zhang et al. [50]. Briefly, 100 mg lyophilized sample was homogenized in 1 mL 0.1% (*w*/*v*) TCA solution. The homogenate was centrifuged at 13,000 rpm for 10 min. Then, 1 mL supernatant was added to 4 mL 20% TCA containing 0.5% thiobarbituric acid (TBA) and incubated for 30 min at 96 °C. Immediately, tubes were transferred into ice bath to stop the reaction. Solution was centrifuged again at 10,00 rpm for 5 min and the absorbance of the supernatant was recorded at 532 nm. The standard curve was generated from MDA standard. The concentration of MDA was calculated from the absorbance knowing the calibration curve. 

Water-soluble phenols content in stem and leaves of alfalfa was measured using the Folin–Ciocalteau reagent, following the method of Box [45]. Gallic acid was used as the standard and the concentration of water-soluble phenols was expressed as gallic acid equivalents per g dry weight. 

### 4.9. Data Analysis

Dependent variables were checked for normality and homoscedasticity and transformed as necessary. Data analysis was performed using Microsoft Excel 2010 and the SPSS 13.0 software package (SPSS Inc., Chicago, IL, USA). The analysis of variance using two-way ANOVA was conducted between Se treatments and harvests, while one-way ANOVA was applied to evaluate the differences among Se treatments within the same harvest. Separation of means was performed by post-hoc test (Tukey’s test), and significant differences were accepted at the levels *p* < 0.05, 0.01, and 0.001. The data were presented as mean ± standard deviation (*n* = 9). A principal component analysis (PCA) was run to define the interaction between the measured morphological, chemical, and biochemical properties of alfalfa plants. The KMO and Bartlett’s sphericity test was applied to check the validity of PCA to the data derived from the present study. Spearman coefficient was run to investigate the non-parametric correlation between alfalfa traits. 

## 5. Conclusions

The present study aimed to investigate the biofortification of alfalfa with Se using ionic, i.e., Se (IV) and Se (VI), and elemental Se forms. However, the uptake dynamics of Se within four consecutive harvests is of most interest since this paper, to our knowledge, is the first to report Se-fortified multi-harvest forage crop. Our results showed that for long-term biofortification red Se^0^ and Se (IV) are much better than Se (VI). Almost a constant total Se content in stem and leaf of alfalfa was measured within the four harvests for those plants treated with red Se^0^ or Se (IV). Moreover, the application of red Se^0^ at the rate of 10 mg L^−1^ exhibited promising results not only regarding the total Se content but also concerning the Se forms in plant tissues. However, more investigations on synthesis of red Se^0^ are needed to have it with a diameter below 25 nm since nanoparticles with a short diameter and large surface area have different physicochemical behavior than the larger ones. Moreover, the possible conversion of red Se^0^ to Se (IV) in soil or on root surface requires more explanation alongside focusing on the plant’s response to the application, uptake, accumulation, and transformation of red Se^0^ within plant tissues into organic Se forms. 

## Figures and Tables

**Table 1:** Selenomethionine (SeMet) and selenate (Se (VI)) content in stem and leaves of alfalfa grown on different selenium (Se) forms and concentrations within three harvests.

Treatments	Water Extract											
	Stem						Leaves					
	SeMet (mg kg^−1^)			Se (VI) (mg kg^−1^)			SeMet (mg kg^−1^)			Se (VI) (mg kg^−1^)		
	1st	2nd	4th	1st	2nd	4th	1st	2nd	4th	1st	2nd	4th
10 Se (VI)	2.2	0.9	0.5	79.5	32.3	4.3	8.2	1.5	1.2	901.0	141.0	10.7
50 Se (IV)	1.7	1.4	1.1	89.0	22.4	5.2	2.3	1.9	4.2	336.0	65.8	20.5
50 red Se^0^	0.6	0.3	0.5	6.5	0.7	2.7	1.8	0.6	1.4	51.7	3.9	1.5
	Enzyme Extract											
	Stem						Leaves					
	SeMet (mg kg^−1^)			Se (VI) (mg kg^−1^)			SeMet (mg kg^−1^)			Se (VI) (mg kg^−1^)		
	1st	2nd	4th	1st	2nd	4th	1st	2nd	4th	1st	2nd	4th
10 Se (VI)	18.1	14.3	4.8	8.1	6.0	0.8	71.4	41.0	24.8	86.8	18.1	2.6
50 Se (IV)	17.7	12.3	8.5	13.0	5.2	0.8	56.9	34.9	22.4	30.8	12.9	2.9
50 red Se^0^	7.1	4.1	11.9	1.1	Nd ^¥^	nd	20.2	13.1	7. 6	5.4	0.6	nd

^¥^ not detected.

**Table 2:** Variations in some biochemical traits in alfalfa grown on different selenium (Se) forms (Se (VI), Se (IV), and red Se^0^) and concentrations (1, 10, and 50 mg kg^−1^ for ionic forms and 10 and 50 mg L^−1^ for elemental form) during four consecutive harvests.

	Malondialdehyde (nmol g^−1^)				Water-Soluble Phenols (µg g^−1^)				Peroxidase Activity (U mL^−1^ min^−1^ g^−1^ DW)			
	Stem				Stem				Stem			
	1st Harvest	2nd Harvest	3rd Harvest	4th Harvest	1st Harvest	2nd Harvest	3rd Harvest	4th Harvest	1st Harvest	2nd Harvest	3rd Harvest	4th Harvest
Control	20.5 ± 0.36 cd	13.4 ± 0.29 a	10.5 ± 0.00 d	11.6 ± 0.40 ab	45.5 ± 0.58 e	51.5 ± 2.38 b	41.8 ± 0.50 d	48.3 ± 2.50 cd	21.7 ± 0.08 e	39.7 ± 0.29 e	35.9 ± 0.32 c	24.0 ± 0.17 e
1 Se (VI)	24.9 ± 0.47 a	11.6 ± 0.55 b	10.3 ± 0.10 e	11.3 ± 0.35 ab	54.3 ± 0.50 b	44.0 ± 2.16 c	40.5 ± 0.58 de	46.5 ± 2.89 d	40.4 ± 0.09 bc	45.3 ± 0.26 c	44.5 ± 0.26 a	44.6 ± 0.26 b
10 Se (VI)	24.8 ± 0.25 ab	12.1 ± 0.72 b	9.9 ± 0.06 f	11.2 ± 0.38 ab	67.5 ± 0.58 a	65.3 ± 1.50 a	36.8 ± 0.50 f	52.0 ± 1.41 c	30.5 ± 0.08 d	12.2 ± 0.26 h	8.2 ± 0.26 g	25.9 ± 0.18 d
1 Se (IV)	19.8 ± 0.32 de	11.9 ± 0.31 b	11.3 ± 0.06 b	10.6 ± 0.62 b	46.3 ± 0.50 de	52.5 ± 2.38 b	41.8 ± 0.50 d	61.0 ± 2.94 b	67.5 ± 0.05 a	50.4 ± 0.26 b	36.0 ± 0.25 c	53.8 ± 0.25 a
10 Se (IV)	23.6 ± 0.50 b	12.0 ± 0.21 b	11.6 ± 0.06 a	12.0 ± 0.40 a	47.3 ± 0.50 cd	48.0 ± 2.94 bc	39.8 ± 0.50 e	56.8 ± 0.50 b	40.4 ± 0.10 bc	42.0 ± 0.34 d	43.5 ± 0.31 b	35.0 ± 0.17 c
50 Se (IV)	25.4 ± 0.38 a	11.4 ± 0.35 b	10.6 ± 0.00 d	11.7 ± 0.66 ab	47.8 ± 0.50 c	51.0 ± 1.63 b	46.8 ± 0.50 b	77.3 ± 1.50 a	39.3 ± 0.14 c	14.6 ± 0.33 g	11.3 ± 0.36 f	20.0 ± 0.36 g
10 red Se^0^	18.6 ± 0.65 e	12.5 ± 0.15 ab	10.4 ± 0.06 de	11.3 ± 0.35 ab	47.3 ± 0.50 cd	49.5 ± 2.38 b	45.3 ± 0.96 c	76.8 ± 1.26 a	28.9 ± 0.14 de	54.2 ± 0.25 a	34.5 ± 0.31 d	53.3 ± 0.31 g
50 red Se^0^	21.7 ± 0.46 c	12.0 ± 0.42 b	10.9 ± 0.10 c	11.3 ± 0.46 ab	48.0 ± 0.82 c	49.0 ± 2.16 bc	48.5 ± 0.58 a	72.8 ± 1.71 a	48.5 ± 0.17 b	18.1 ± 0.24 f	24.3 ± 0.25 e	21.2 ± 0.25 f
F-test (Two-ways)															
Treatment	***				***				***			
Harvest	***				***				***			
Treatment × Harvest	***				***				***			
	Leaf				Leaf				Leaf			
	1st harvest	2nd harvest	3rd harvest	4th harvest	1st harvest	2nd harvest	3rd harvest	4th harvest	1st harvest	2nd harvest	3rd harvest	4th harvest
Control	16.9 ± 0.25 c	18.4 ± 0.55 a	14.7 ± 0.40 d	18.4 ± 0.59 cd	78.0 ± 1.73 b	150.8 ± 0.50 c	144.8 ± 0.96 a	104.8 ± 0.50 c	49.8 ± 0.13 c	24.8 ± 0.31 d	18.6 ± 0.26 d	47.5 ± 0.33 c
1 Se (VI)	18.2 ± 0.23 ab	16.1 ± 1.16 b	15.5 ± 0.42 cd	17.1 ± 0.62 d	133.5 ± 1.00 a	143.5 ± 0.58 e	138.3 ± 0.96 b	74.0 ± 0.82 f	39.1 ± 0.13 e	29.0 ± 0.36 c	20.6 ± 0.31 c	32.4 ± 0.29 e
10 Se (VI)	17.9 ± 0.40 abc	15.7 ± 0.78 b	15.1 ± 0.68 d	18.3 ± 0.51 cd	139.3 ± 0.50 a	145.8 ± 0.96 d	142.8 ± 0.96 a	114.8 ± 0.96 b	63.2 ± 0.20 a	11.6 ± 0.17 a	5.0 ± 0.19 h	15.3 ± 0.32 h
1 Se (IV)	17.7 ± 0.47 bc	15.8 ± 0.53 b	17.8 ± 0.21 b	18.6 ± 0.36 bcd	147.8 ± 0.50 a	153.8 ± 0.50 b	116.0 ± 1.15 e	75.0 ± 1.15 f	55.7 ± 0.13 b	42.4 ± 0.28 a	53.1 ± 0.21 a	49.5 ± 0.26 b
10 Se (IV)	18.3 ± 0.35 ab	18.9 ± 0.56 a	16.9 ± 0.57 bc	21.2 ± 0.31 a	127.3 ± 0.50 a	150.5 ± 0.58 c	122.3 ± 0.50 c	80.8 ± 0.96 e	43.3 ± 0.28 d	29.0 ± 0.22 c	15.3 ± 0.22 f	45.6 ± 0.30 d
50 Se (IV)	17.9 ± 0.66 abc	18.2 ± 0.64 a	15.6 ± 0.38 cd	19.7 ± 0.83 abc	144.8 ± 0.96 a	225.3 ± 0.50 a	61.5 ± 0.58 g	80.0 ± 0.82 e	33.6 ± 0.13 g	12.7 ± 0.17 f	7.3 ± 0.22 g	23.4 ± 0.31 f
10 red Se^0^	18.6 ± 0.44 ab	17.5 ± 0.00 ab	17.1 ± 0.52 b	20.2 ± 0.65 ab	119.8 ± 0.96 a	69.5 ± 0.58 g	70.0 ± 0.82 f	95.5 ± 1.00 d	38.8 ± 0.15 e	36.1 ± 0.31 b	17.5 ± 0.22 e	61.5 ± 0.37 a
50 red Se^0^	19.0 ± 0.49 a	18.4 ± 0.46 a	19.4 ± 0.53 a	20.0 ± 0.78 abc	140.5 ± 0.58 a	89.8 ± 0.13 f	119.0 ± 0.82 d	143.5 ± 0.58 a	37.6 ± 0.18 f	14.3 ± 0.17 e	21.1 ± 0.22 b	17.8 ± 0.28 g
F-test (Two-ways)												
Treatment	***				***				***			
Harvest	***				***				***			
Treatment × Harvest	***				***				***			

Data presented are mean ± SD (*n* = 9). Means in the same column and within the same year followed by different letters are significant according to Tukey’s test (*p* ≤ 0.05). *** significant according to Tukey’s test (*p* ≤ 0.001).

**Table 3:** Morphological traits of alfalfa grown on different selenium (Se) forms (Se (VI), Se (IV), and red Se^0^) and concentrations (1, 10, and 50 mg kg^−1^ for ionic forms and 10 and 50 mg L^−1^ for elemental form) during four consecutive harvests.

	Shoot Length (cm)				Shoot DM (g plant^−1^)			
	1st Harvest	2nd Harvest	3rd Harvest	4th Harvest	1st Harvest	2nd Harvest	3rd Harvest	4th Harvest
Control	38.9 ± 4.75 a	32.2 ± 3.21 cd	41.4 ± 5.20 b	45.9 ± 6.42 a	0.66 ± 0.26 a	0.35 ± 0.10 b	0.90 ± 0.46 ab	0.74 ± 0.22 a
1 Se (VI)	39.0 ± 6.93 a	47.6 ± 5.53 a	51.5 ± 9.44 ab	44.5 ± 5.30 ab	0.40 ± 0.17 ab	1.05 ± 0.46 a	0.88 ± 0.30 ab	0.65 ± 0.24 ab
10 Se (VI)	29.8 ± 3.46 b	29.8 ± 4.98 d	45.8 ± 5.22 b	39.5 ± 6.13 abc	0.27 ± 0.11 b	0.38 ± 0.13 b	0.65 ± 0.25 b	0.40 ± 0.09 bc
1 Se (IV)	34.9 ± 5.43 ab	40.3 ± 5.86 ab	48.6 ± 4.53 ab	39.5 ± 4.90 abc	0.55 ± 0.25 ab	0.68 ± 0.36 b	0.98 ± 0.31 ab	0.61 ± 0.19 ab
10 Se (IV)	38.7 ± 5.99 a	40.1 ± 4.42 b	58.4 ± 7.24 a	41.5 ± 4.93 abc	0.61 ± 0.31 a	0.59 ± 0.23 b	1.26 ± 0.50 a	0.49 ± 0.15 abc
50 Se (IV)	33.1 ± 4.13 ab	35.5 ± 7.05 bcd	48.1 ± 5.74 ab	35.4 ± 3.95 c	0.39 ± 0.16 ab	0.38 ± 0.21 b	0.69 ± 0.29 b	0.27 ± 0.10 c
10 red Se^0^	34.2 ± 2.86 ab	37.6 ± 5.15 bc	50.1 ± 8.92 ab	36.6 ± 4.43 bc	0.45 ± 0.14 ab	0.44 ± 0.18 b	0.73 ± 0.27 b	0.44 ± 0.17 bc
50 red Se^0^	35.2 ± 4.13 ab	40.9 ± 5.28 ab	41.3 ± 5.33 b	39.0 ± 9.61 abc	0.49 ± 0.25 ab	0.60 ± 0.21 b	0.77 ± 0.30 b	0.57 ± 0.24 ab
F-test (Two-ways)								
Treatment	***				***			
Harvest	***				***			
Treatment × Harvest	***				***			

Data presented are mean ± SD (*n* = 9). Means in the same column and within the same year followed by different letters are significant according to Tukey’s test (*p* ≤ 0.05). *** significant according to Tukey’s test (*p* ≤ 0.001).

**Table 4:** Rotated loading values for the first five PCs from the measured properties of alfalfa grown on different selenium (Se) forms (Se (VI), Se (IV), and red Se^0^) and concentrations (1, 10, and 50 mg kg^−1^ for ionic forms and 10 and 50 mg L^−1^ for elemental form) during four consecutive harvests (in bold significant values *p* < 0.01).

Component Matrix ^(a)^					
	Component				
	1	2	3	4	5
Harvests	**−0.605**	**0.551**	−0.304	−0.199	0.326
Treatments	0.140	0.451	−0.148	−0.320	**−0.694**
Se_Stem	**0.754**	−0.242	−0.439	−0.144	0.219
Se_Leaf	**0.730**	−0.231	−0.469	−0.160	0.236
MDA_Stem	**0.779**	−0.312	0.389	−0.124	
MDA_Leaf	0.327	**0.769**	0.204	−0.104	−0.250
Protein_Stem	0.233	0.277	0.185	**0.774**	−0.102
Protein_Leaf	**−0.610**	0.341	−0.192	0.407	0.200
Phenol_Stem	0.459	**0.719**	−0.203		0.265
Phenol_Leaf	0.311	−0.439	−0.146	0.435	−0.182
POD_Stem			**0.809**		0.140
POD_Leaf	0.397	0.188	**0.723**	−0.122	0.373
Shoot_Length	**−0.848**	−0.219		−0.197	
Shoot_DW	**−0.755**	−0.400	0.175	−0.130	
**Eigenvalue**	4.38	2.46	2.06	1.24	1.08
**Cumulative %**	31.3	48.9	63.9	72.5	80.2

Extraction method: principal component analysis. ^a^ Five components extracted.

## Data Availability

All data are available within the text and Supplementary Materials.

## Supplementary Materials

The following are available online at https://www.mdpi.com/article/10.3390/plants10071277/s1. Figure S1: HPLC-ICP-MS chromatograms of selenium (Se) forms in three different solutions, i.e., 10 and 50 mg kg^−1^ Se (VI) and Se (IV), respectively, and 50 mg L-1 red Se^0^ within three harvests, i.e., 1st, 2nd, and 4th harvests. U1 and U2 denote unknown selenocompounds. Table S1: Pearson correlation between the measured parameters of alfalfa grown in presence of different Se forms (Se (VI, Se (IV), and red Se^0^) and concentrations (1, 10, and 50 mg kg^−1^ for the ionic forms and 10 and 50 mg L^−1^ for elemental form). Table S2: Total variance explained for alfalfa traits grown on different selenium (Se) forms (Se (VI), Se (IV), and red Se^0^) and concentrations (1, 10, and 50 mg kg^−1^ for ionic forms and 10 and 50 mg L^−1^ for elemental form) during four consecutive harvests. Table S3: Physicochemical characteristics of the experimental soil.
