# Supplementary material for: Uptake Dynamics of Ionic and Elemental Selenium Forms and Their Metabolism in Multiple-Harvested Alfalfa (Medicago sativa L.)
Source: Plants (Basel). 2021 Jun 23;10(7):1277. doi: 10.3390/plants10071277 (PMC8309208; doi:10.3390/plants10071277)
Supplement: Supplementary file 1 [file plants-10-01277-s001.zip › plants-1265141-supplementary.pdf]

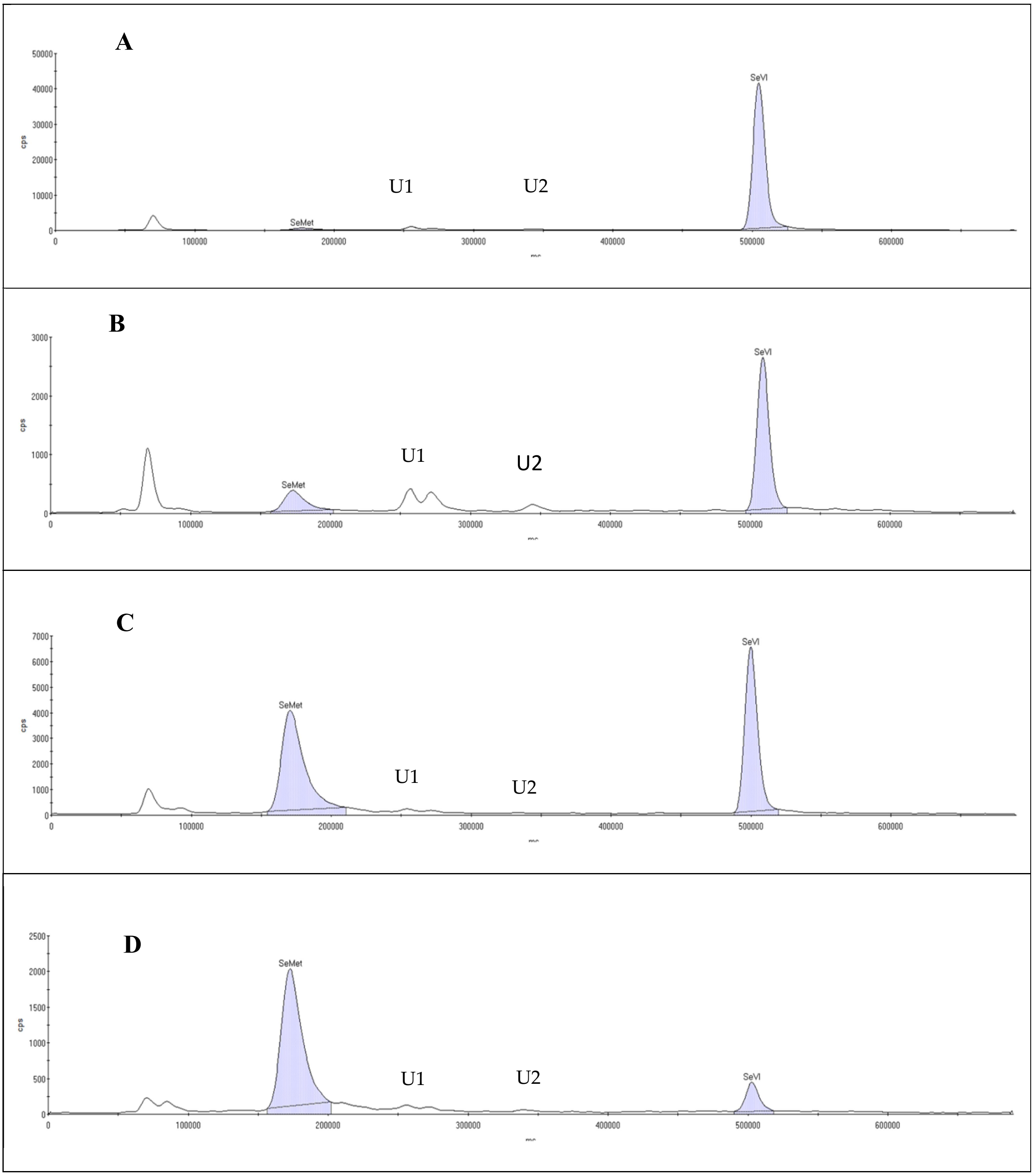

Figure S1. A: (SAX)–ICP-MS chromatogram of the water extracted stem treated by 50 mg Se kg^{-1} Se (IV) from the 1st harvest; B: (SAX)–ICP-MS chromatogram of the water extracted stem treated by 50 mg Se kg^{-1} Se (IV) from the 4st harvest; C: (SAX)–ICP-MS chromatogram of the enzymatically extracted stem treated by 50 mg Se kg^{-1} Se (IV) from the 1st harvest; D: SAX)–ICP-MS chromatogram of the enzymatically extracted stem treated by 50 mg Se kg^{-1} Se (IV) from the 4st harvest. U1 and U2 denote unknown seleno-compounds.

Table S1. Pearson correlation between the measured parameters of alfalfa grown in presence of different Se forms (Se (VI, Se (IV) and red Se^0) and concentrations (1, 10 and 50 mg kg^{-1} for the ionic forms and 10 and 50 mg L^{-1} for elemental form)

		Harvests	Treatment	Se_ Stem	Se_ Leaf	MDA_ Stem	MDA_ Leaf	Protein_ Stem	Protein_ Leaf	Phenol_ Stem	Phenol_ Leaf	POD_ Stem	POD_ Leaf	Shoot_ Length	Shoot_ DW
Harvests	Correlation	1	**.000**	**-.364(*)**	-.330	-.778(**)	.184	-.251	.525(**)	.265	-.401(*)	-.154	-.213	.427(*)	.212
	Sig. (2-tailed)		1.000	.040	.065	.000	.314	.166	.002	.142	.023	.401	.241	.015	.244
Treatments	Correlation	.000	1	.049	.053	-.025	.486(**)	-.013	-.020	.255	-.132	-.050	-.130	-.103	-.178
	Sig. (2-tailed)	1.000		.788	.772	.893	.005	.945	.912	.159	.472	.785	.479	.574	.329
Se_Stem	Correlation	**-.364(*)**	.049	1	.992(**)	.482(**)	-.044	-.024	-.376(*)	.297	.303	-.259	.076	-.485(**)	-.450(**)
	Sig. (2-tailed)	.040	.788		.000	.005	.811	.896	.034	.099	.092	.153	.679	.005	.010
Se_Leaf	Correlation	-.330	.053	**.992(**)**	1	.446(*)	-.061	-.037	-.358(*)	.303	.272	-.288	.062	-.462(**)	-.439(*)
	Sig. (2-tailed)	.065	.772	.000		.011	.739	.841	.044	.092	.132	.110	.736	.008	.012
MDA_Stem	Correlation	**-.778(**)**	-.025	**.482(**)**	**.446(*)**	1	.148	.128	-.709(**)	.024	.182	.207	.484(**)	-.496(**)	-.387(*)
	Sig. (2-tailed)	.000	.893	.005	.011		.419	.484	.000	.898	.320	.256	.005	.004	.028
MDA_Leaf	Correlation	.184	**.486(**)**	-.044	-.061	.148	1	.272	-.113	.550(**)	-.143	.098	.368(*)	-.346	-.444(*)
	Sig. (2-tailed)	.314	.005	.811	.739	.419		.133	.538	.001	.434	.592	.038	.052	.011
Protein_Stem	Correlation	-.251	-.013	-.024	-.037	.128	.272	1	.179	.214	.089	-.012	.187	-.259	-.242
	Sig. (2-tailed)	.166	.945	.896	.841	.484	.133		.327	.239	.626	.949	.306	.153	.182
Protein_Leaf	Correlation	**.525(**)**	-.020	**-.376(*)**	**-.358(*)**	**-.709(**)**	-.113	.179	1	.003	-.184	-.007	-.259	.374(*)	.234
	Sig. (2-tailed)	.002	.912	.034	.044	.000	.538	.327		.988	.314	.971	.152	.035	.198
Phenol_Stem	Correlation	.265	.255	.297	.303	.024	**.550(**)**	.214	.003	1	-.105	-.071	.245	-.548(**)	-.603(**)
	Sig. (2-tailed)	.142	.159	.099	.092	.898	.001	.239	.988		.568	.701	.177	.001	.000
Phenol_Leaf	Correlation	**-.401(*)**	-.132	.303	.272	.182	-.143	.089	-.184	-.105	1	-.027	-.151	-.272	-.068
	Sig. (2-tailed)	.023	.472	.092	.132	.320	.434	.626	.314	.568		.885	.409	.132	.712
POD_Stem	Correlation	-.154	-.050	-.259	-.288	.207	.098	-.012	-.007	-.071	-.027	1	.574(**)	.012	.140
	Sig. (2-tailed)	.401	.785	.153	.110	.256	.592	.949	.971	.701	.885		.001	.947	.443
POD_Leaf	Correlation	-.213	-.130	.076	.062	**.484(**)**	**.368(*)**	.187	-.259	.245	-.151	**.574(**)**	1	-.296	-.164
	Sig. (2-tailed)	.241	.479	.679	.736	.005	.038	.306	.152	.177	.409	.001		.100	.371
Shoot_Length	Correlation	**.427(*)**	-.103	**-.485(**)**	**-.462(**)**	**-.496(**)**	-.346	-.259	**.374(*)**	**-.548(**)**	-.272	.012	-.296	1	.863(**)
	Sig. (2-tailed)	.015	.574	.005	.008	.004	.052	.153	.035	.001	.132	.947	.100		.000
Shoot_DW	Correlation	.212	-.178	**-.450(**)**	**-.439(*)**	**-.387(*)**	**-.444(*)**	-.242	.234	**-.603(**)**	-.068	.140	-.164	**.863(**)**	1
	Sig. (2-tailed)	.244	.329	.010	.012	.028	.011	.182	.198	.000	.712	.443	.371	.000	

* Correlation is significant at the 0.05 level (2-tailed).
** Correlation is significant at the 0.01 level (2-tailed).
n= 32

Table S2. Total variance explained for alfalfa traits grown on different selenium (Se) forms (Se (VI), Se (IV) and red Se^0) and concentrations (1, 10 and 50 mg kg^{-1} for ionic forms and 10 and 50 mg L^{-1} for elemental form) during four consecutive harvests

Component	Initial Eigenvalues			Extraction Sums of Squared Loadings			Rotation Sums of Squared Loadings		
	Total	% of Variance	Cumulative %	Total	% of Variance	Cumulative %	Total	% of Variance	Cumulative %
1	4.384	31.312	31.312	4.384	31.312	31.312	3.267	23.333	23.333
2	2.455	17.539	48.851	2.455	17.539	48.851	2.789	19.921	43.253
3	2.064	14.741	63.591	2.064	14.741	63.591	1.960	13.998	57.251
4	1.244	8.887	72.478	1.244	8.887	72.478	1.791	12.793	70.044
5	1.077	7.696	80.174	1.077	7.696	80.174	1.418	10.131	80.174
6	.773	5.521	85.695						
7	.721	5.150	90.845						
8	.547	3.906	94.751						
9	.272	1.943	96.694						
10	.198	1.417	98.111						
11	.150	1.072	99.183						
12	.071	.507	99.689						
13	.037	.266	99.955						
14	.006	.045	100.000						

Table S3. Physicochemical characteristics of the experimental soil

Property	Value
pH	7.78 ± 0.03
Electrical conductivity (EC_e, dS m^{-1})	0.48 ± 0.01
Soil organic matter (g kg^{-1})	18.69 ± 0.14
Saturation % (mL H_2O)/ 100 g soil)	36.0 ± 1.62
Macro and micro-elements (mg kg^{-1})	
N	0.155 ± 0.008
P	2015 ± 135
K	5860 ± 245
Na	177 ± 21
Ca	17111 ± 532
Al	18829 ± 545
As	3.96 ± 0.15
B	16.5 ± 1.25
Ba	115 ± 7.2
Cd	nd*
Co	8.46 ± 0.97
Cr	37.9 ± 1.55
Cu	18.6 ± 2.07
Fe	19453 ± 623
Mg	5156 ± 119
Mn	599 ± 12
Mo	nd
Ni	24.3 ± 1.5
Pb	11.8 ± 0.9
S	220 ± 5.6
Sr	58.4 ± 1.8
Zn	65.6 ± 2.1

* not detected
